# Clinodiside A, an active metabolite in Clinopodii herba, alleviates dextran-sulfate-sodium-induced ulcerative colitis

**DOI:** 10.3389/fphar.2026.1828440

**Published:** 2026-06-17

**Authors:** Xingxing Yang, Yujia Fan, Qiong Lin, Jinxin Ni, Xinsheng Tan, Jiajun Xu, Min Sun

**Affiliations:** School of Biological and Food Engineering, Hefei Normal University, Hefei, Anhui, China

**Keywords:** buddlejasaponin IVb, Clinodiside A, Clinopodii herba, dextran sulfate sodium, ulcerative colitis

## Abstract

**Introduction:**

Clinodiside A is a natural bioactive component of Clinopodii herba, a genuine regional herb from Anhui Province (China) with multiple biological activities. This study aimed to explore the therapeutic potential of Clinodiside A in ulcerative colitis (UC).

**Methods:**

Dextran sulfate sodium (DSS) was used to establish UC models both *in vivo* and *in vitro*. The models were treated with Clinodiside A. Parameters including body weight, disease activity index (DAI) score, colon length, and inflammation were assessed. Iron overload, lipid peroxidation, and intestinal epithelial repair were also evaluated. *In vitro* experiments using HCT116 cells examined cell damage, motility, and recovery.

**Results:**

Clinodiside A alleviated DSS-induced UC consequences, including weight loss, increased DAI score, colon shortening, and inflammation. These effects were associated with reduced iron overload, reduced lipid peroxidation, and enhanced intestinal epithelial repair. In HCT116 cells, Clinodiside A ameliorated damage, enhanced motility, and promoted recovery of the intestinal epithelium.

**Discussion:**

Our research validates the therapeutic and protective effects of Clinodiside A from Clinopodii herba on UC and expands its application potential.

## 1 Introduction

Ulcerative colitis (UC) is one of the main subtypes of inflammatory bowel disease (IBD) that is characterized by immune system dysfunction (Zhao et al., 2019); it is a chronic and non-specific inflammatory disease of the large intestine featuring continuous and diffuse inflammation of the colorectal mucosa. The pathological changes in UC are primarily confined to the mucosal and submucosal layers, and these changes can affect the rectum, the distal colon, or even the entire colon. As of 2023, approximately 5 million people were known to be affected by UC globally; this condition often presents with abdominal pain, rectal bleeding, and chronic or subacute diarrhea (Le Berre et al., 2023), along with other symptoms such as arthritis, inflamed bile ducts, or eye inflammation. UC can ultimately lead to complications, such as colorectal cancer, depression, anxiety, sleep disturbances, and sexual dysfunction, all of which pose a considerable public health burden (Voelker, 2024). Notably, the pathogenesis of UC remains unclear, and there is a lack of specific therapeutic drugs (Du and Ha, 2020). Anti-inflammatory therapies, such as those involving mesalazine and glucocorticoids, represent the primary treatment approach for UC; furthermore, fecal microbiota transplantation has been found to offer certain therapeutic benefits and has shown efficacy in some cases (Marsool et al., 2023).

Clinopodii herba, also known as “Duanxueliu,” is a well-recognized herba that has been officially documented in the Pharmacopoeia of the People’s Republic of China (2020 edition). It comprises the dried aerial parts of perennial herbaceous plants from the *Lamiaceae* family, specifically *Clinopodium polycephalum* (Vaniot) C.Y.Wu &Hsuan or *Clinopodium chinense* (Benth.) O. Kuntze. Clinopodii herba is an authentic regional herba from Anhui Province (China) that was included in the “Top Ten Anhui Medicines” in 2016. Clinopodii herba exhibits various pharmacological effects, including hemostatic, anti-inflammatory, antibacterial, and uterotonic properties (Cheng et al., 2007). Given the hallmark features of UC, namely chronic colorectal inflammation and rectal bleeding, the anti-inflammatory and hemostatic activities of Clinopodii herba are directly aligned with the disease pathology; this provides a scientific rationale for investigating this herba in the disease model of UC. Clinodiside A, also known as buddlejasaponin IVb, is the active metabolite in Clinopodii herba; it has been widely reported to possess a range of pharmacological activities, including anti-inflammatory (Sun et al., 2022), antioxidant (Bektašević et al., 2022), anti-Parkinson’s (Li et al., 2024; Li et al., 2026), and neuroprotective (Wang et al., 2018) effects.

Ferroptosis is a novel form of cell death characterized by iron-dependent lipid peroxidation; it is governed by multiple biological processes, including iron metabolism, redox homeostasis, and lipid and amino acid metabolism (Dixon et al., 2012). The dysregulation of iron metabolism homeostasis in the body leads to ferrous (Fe^2+^) iron overload, which in turn generates lipid peroxidation products, such as lipid reactive oxygen species (ROS) and malondialdehyde (MDA), thereby initiating ferroptosis (Galy et al., 2024; Yao et al., 2024; Zhang, 2024). Recent studies have reported that ferroptosis is a critical contributor to UC. In particular, Chen et al. (2020) found that suppression of ferroptosis could effectively ameliorate dextran sulfate sodium (DSS)-induced UC, which is involved in blocking the Nrf2/HO-1 signaling pathway. Emerging evidence suggests that Clinodiside A or buddlejasaponin IVb can regulate iron metabolism; for instance, Xu et al. (2020) discovered that ferroptosis contributes to UC through endoplasmic reticulum (ER)-stress-mediated intestinal epithelial cell (IEC) death and that NF-κB p65 phosphorylation suppresses ER-stress-mediated IEC ferroptosis to alleviate UC. Additionally, buddlejasaponin IVb was shown to suppress iron-overload-mediated dopaminergic neuron ferroptosis and improve motor dysfunction in Parkinson’s disease by targeting DEAD-box helicase 17 (Li et al., 2026). Notably, *C. chinense* Kuntze was shown to ameliorate DSS-induced UC in mice through systemic inflammation reduction and metabolism regulation (Wang et al., 2023). Similarly, Wu et al. (2024) demonstrated that buddlejasaponin IVb alleviates DSS-induced UC through Nrf2/GPX4 pathway activation and gut microbiota modulation. Therefore, we hypothesize that Clinodiside A, which is the active metabolite in *C. chinense*, can alleviate DSS-induced UC by modulating iron levels, attenuating lipid peroxidation, and promoting intestinal epithelial migration and repair. In the present study, we investigated the anti-UC effects of Clinodiside A by establishing DSS-induced UC models both *in vivo* and *in vitro*; furthermore, we explored the underlying mechanisms by specifically focusing on regulation of the iron level and enhancement of the IEC motility, thereby promoting intestinal mucosal repair.

## 2 Methods

### 2.1 Animal experimental protocols

A total of 60 six-week-old male C57BL/6J mice were obtained from Beijing Vital River Laboratory Animal Technology Limited (Beijing, China). The mice were housed in plastic cages under controlled environmental conditions as follows: temperature range of 22 °C–24 °C, relative humidity of 50%–60%, and 12-h light/dark cycle. Water and food were provided *ad libitum*. Following one week of adaptive feeding, the mice were randomly assigned to five groups as follows: control, model, low-dose (10 mg/kg), high-dose (40 mg/kg), and mesalazine or 5-aminosalicylic acid (5-ASA; positive control). All protocols were approved by the Ethics and Humane Committee of Hefei Normal University (no. 2024LLSP017) and were performed in compliance with the National Institutes of Health Guide for the Care and Use of Laboratory Animals (NIH publication no. 8023, revised 1978).

Clinodiside A (analytical grade reference standard; purity ≥ 98% by high-performance liquid chromatography (HPLC); CAS no. 152580-79-5; batch no. DST240306-048) was purchased from Desite Biological Co., Ltd. (Chengdu, China); this powder was stored at 4 °C and away from light prior to use. Chemically, Clinodiside A is a pentacyclic triterpenoid saponin that is practically insoluble in water, soluble in dimethyl sulfoxide and methanol, and stable under dark conditions at room temperature. For *in vivo* administration, it was prepared as a homogeneous suspension in 0.5% CMC-Na for gavage. The low and high doses of Clinodiside A (10 mg/kg and 40 mg/kg, respectively) were determined through preliminary gradient dose screening experiments in mice, which demonstrated that both doses were effective and non-lethal for anti-UC activity. The low-dose and high-dose groups were thus administered 10 mg/kg and 40 mg/kg of Clinodiside A, respectively, by gavage at a volume of 10 mL/kg/d. The control and model groups received equal volumes of 0.5% CMC-Na by gavage daily, while the mesalazine (5-ASA) group was given 200 mg/kg of mesalazine by gavage each day as a positive control. The mesalazine dose was selected based on previous studies; the 200 mg/kg dose used in this work is considerably lower than the mouse-equivalent dose (800 mg/kg/d) derived from the standard human clinical dose (4.8 g/d) (Li et al., 2015). Furthermore, previous chronic administration studies have confirmed that 200 mg/kg of 5-ASA does not lead to significant drug accumulation or potential toxicity in major organs, including the liver, lungs, and brain, thereby ruling out the possibility of toxic artifacts (Li et al., 2015; Jin et al., 2024).

**FIGURE 1:**
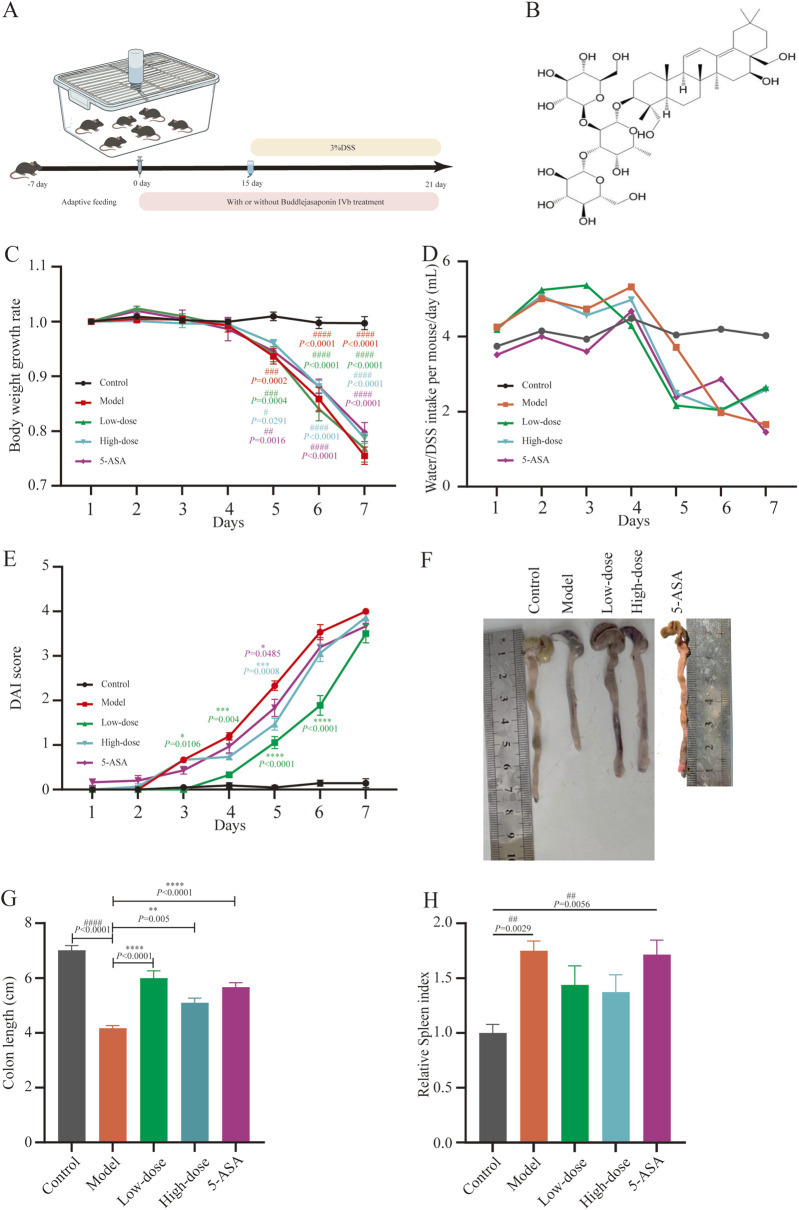
Clinodiside A alleviates dextran sulfate sodium (DSS)-induced ulcerative colitis (UC): **(A)** schematic illustration of the experimental procedures; **(B)** structural formula of Clinodiside A; **(C)** changes in the bodyweights of the mice used in the study during DSS induction; **(D)** daily water consumption by the mice during DSS induction; **(E)** daily disease activity index (DAI) scores of the mice during DSS induction; representative **(F)** pictures of the colon tissues and **(G)** colon lengths in all study groups; **(H)** relative spleen index values in all study groups. ^#^ *p* < 0.05 (vs. control) and *p < 0.05 (vs. model).

All mice underwent continuous treatment administration for 21 d. On day 15, the experimental groups were administered a 3% solution of DSS (MP Biomedicals, catalog no. 160110) *ad libitum* for 7 d to induce the UC model, whereas the control group was given pure drinking water over the same period (Figure 1A). Throughout the modeling period, the bodyweight values of the mice along with their food and water consumption were recorded daily. The fecal characteristics were observed, and fecal occult blood was assessed using commercial occult blood test strips (YaHua Biotechnology Company, Lanzhou, China). On day 21, blood samples were collected from the mice after euthanization by CO_2_ inhalation. All major organs, including the heart, liver, spleen, lungs, kidneys, and colon, were collected and weighed. A 2-cm segment from the distal colon was obtained from each animal and fixed in 4% paraformaldehyde solution for subsequent hematoxylin and eosin (H&E) staining. Following photographic documentation, the remaining colon tissue was frozen and stored at −80 °C.

### 2.2 Disease activity index

Throughout the DSS administration period, the bodyweight, fecal consistency, and fecal occult blood of the mice were monitored daily. The DAI score was evaluated using a previously established protocol (Yang et al., 2023) by combining three parameters, namely the percentage of weight loss, diarrhea, and hematochezia, each of which is scored on a four-point scale. The fecal occult blood was detected by the colloidal gold method using commercially available occult blood test strips.

### 2.3 Enzyme-linked immunosorbent assay

The serum concentrations of specific pro-inflammatory cytokines (TNF-α, IL-1β, and IL-6) were measured using commercial ELISA kits (Hua Biotechnology, Hangzhou, China). Prior to usage, all kits were allowed to equilibrate to room temperature for 20 min after removal from storage at 4 °C. The assay was performed as follows: (1) blank wells were left empty, while 50 µL of serially diluted standards were added to the standard wells, and 50 µL of the fresh serum was added to the sample wells; (2) horseradish-peroxidase-labeled detection antibody (100 µL) was added to each well except the blanks; (3) the plate was sealed and incubated at 37 °C for 1.5 h; (4) the liquid was discarded, and the wells were washed five times each with 350 µL of the wash buffer (1 min per wash), followed by patting dry; (5) substrate A (50 µL) and substrate B (50 µL) were added to each well, and the plate was incubated at 37 °C in the dark for 15 min; (6) the stop solution (50 µL) was added, and the absorbance was measured at 450 nm within 15 min.

### 2.4 H&E staining

After the mice were euthanized, their colons, spleens, hearts, livers, lungs, and kidneys were fixed in 4% paraformaldehyde for 2 d. Subsequently, the paraformaldehyde was removed, and the tissue samples were dehydrated, cleaned, waxed, and embedded in paraffin according to standard protocols. Next, the samples were sliced and stained to obtain the corresponding specimens. Finally, the stained sections were visualized under a light microscope (Olympus, Japan).

### 2.5 Prussian blue staining

The fixed colon segments were dehydrated, cleaned, waxed, embedded in paraffin, and dewaxed. Then, the samples were stained with Perls’ reagent (Sigma) to detect the levels of iron particles. The positive fields were examined using an Olympus microscope and photographed.

### 2.6 Fe^2+^ iron level detection

Approximately 1 mL of the extraction solution (Solarbio, catalog no. BC5415; Elabscience, catalog no. E-BC-K881-M) was added to 0.05 g of the colon tissue or 2 × 10^6^ cells, homogenized in an ice bath, and centrifuged at 12,000 rpm for 10 min at 4 °C, according to the instruction manual. Then, 200 μL of the supernatant and 100 μL of the working solution were collected and added to a 1.5-mL centrifuge tube, mixed thoroughly, and incubated at 37 °C for 10 min. Next, 100 μL of chloroform was added to the mixture and vortexed for 5 min before being centrifuged at 12,000 rpm for 10 min at room temperature. Finally, 200 μL of the upper aqueous phase from this final mixture was transferred to a 96-well plate, and the absorbance was measured at 593 nm.

### 2.7 MDA level detection

Here, approximately 0.05 g of the colon tissue or 2 × 10^6^ treated cells were processed according to the manufacturer protocols (Elabscience, catalog nos. E-BC-F007 and E-BC-K028-M) as follows. First, the samples were homogenized in 1 mL of the extraction solution in an ice bath and centrifuged at 12,000 rpm for 10 min at 4 °C; the supernatant was collected as the test sample. Second, the blank, standard, test, and control tubes were established as follows: 0.1 mL of anhydrous ethanol and 0.1 mL of reagent 1 were added to the blank tube; 0.1 mL of 10 nmol/mL standard and 0.1 mL of reagent 1 were added to the standard tube; 0.1 mL of the test sample and 0.1 mL of reagent 1 were added to the test and control tubes separately. Next, 3 mL of reagent 2 and 1 mL of reagent 3 were mixed and added to the blank, standard, and test tubes, and 1 mL of 50% glacial acetic acid was further added to the blank tube. Third, the centrifuge tubes were capped and mixed after poking a small hole in each cap with a needle; the samples were incubated for 40 min in water at 95 °C. Fourth, the samples were cooled under running water and centrifuged for 10 min at 3,500–4,000 rpm. Finally, the supernatant was collected and absorbance was measured via the optical density (OD) at 532 nm.

### 2.8 Superoxide dismutase activity assay in the colon tissue

The homogenate of tissue or cells was centrifuged at 3,500 rpm for 10–15 min at 4 °C and diluted. Then, the control, control blank, test, and test blank samples were established according to the instruction manual (Solarbio, catalog no. BC0175). Next, we added 20 μL of distilled water and the enzyme working solution separately to the control, 20 μL of distilled water and enzyme dilution to the control blank, 20 μL of the sample and enzyme working solution to the test, and 20 μL of the sample and enzyme dilution to the test blank wells. Lastly, 200 μL of the substrate application solution was added to each group before mixing gently and incubating for 20 min at 37 °C. The ODs of the samples were measured at 450 nm.

### 2.9 Cell culture and cell viability treatment

The human epithelial colon cancer cell line HCT116 is commonly used for *in vitro* studies of drug intervention, cellular injury, and molecular mechanisms in UC (Liang et al., 2025). Based on its wide application in this field, we used HCT116 cells in this study to detect DSS-induced cellular injury and evaluate the drug efficacy. The HCT116 cells were cultured in RPMI 1640 medium (Gibco) containing 10% fetal bovine serum (Sigma) in an incubator at 37 °C under 5% CO_2_. The working concentration of Clinodiside A was determined by a preliminary CCK-8 cytotoxicity screening to ensure that there was no significant inhibition of cell viability; based on the pre-experimental results, 100 μM was selected as the optimal non-toxic and effective concentration. The cells were treated with 1% DSS (Liang et al., 2025; Araki et al., 2006; Montgomery et al., 2025) in the *in vitro* cell model of UC or with 100 μM of Clinodiside A for 24 h. Then, 10 μL of the CCK-8 reagent (APExBIO, catalog no. K1018) was added to each well before incubating at 37 °C for 4 h and measuring the absorbance at 450 nm.

### 2.10 Wound healing assay

The HCT116 cells were seeded in a 6-well plate at 1 × 10^6^ cells/well 24 h in advance. Then, the medium was removed, and a 10 μL pipette tip was used to scratch a “+” in each well; the floating cells were washed away with phosphate-buffered saline, and the washing procedure was repeated thrice. Next, 2 mL of the fresh medium containing 1% DSS was added to each well with or without 100 μM of Clinodiside A, and photos were captured every 24 h.

### 2.11 Statistical analysis

For the *in vitro* experiments, the data were replicated thrice, and all statistical analyses were performed using GraphPad Prism (version 8). The data from the experiments were presented as mean ± standard error of the mean (SEM) values. The data were analyzed using the Shapiro–Wilk test and were found to conform to a normal distribution. If the data were distributed normally, one-way or two-way ANOVA was used to evaluate the differences among groups with *p* < 0.05 being considered as statistically significant; the symbol “#” denotes vs. control and “*” denotes vs. model.

## 3 Results

### 3.1 Clinodiside A alleviated DSS-induced UC in mice

To investigate the protective effects of Clinodiside A (Figure 1B) against UC, we established UC models in mice by administering 3% DSS for 7 d (Figure 1D). Following DSS administration, the mice developed colitis symptoms, including weight loss, diarrhea, and bloody stools. The disease severity was then scored based on these clinical signs. Compared to the control group, the DSS-treated mice exhibited progressive reductions in bodyweight from day 5 following DSS administration. Conversely, treatment with Clinodiside A notably attenuated DSS-induced weight loss, diarrhea, and hematochezia. As shown in Figure 1, Clinodiside A significantly increased the daily bodyweight (Figure 1C) and colon length (Figures 1F,G) while reducing the DAI (Figure 1E) compared to model mice. Although Clinodiside A displayed a favorable trend toward reducing UC-associated splenomegaly, this effect was not statistically significant (Figure 1H).

**FIGURE 2:**
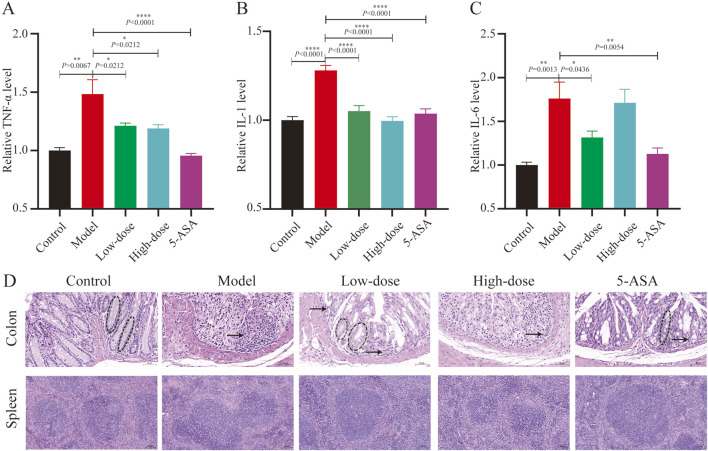
Clinodiside A reduces DSS-induced inflammatory responses associated with UC based on the **(A)** TNF-α, **(B)** IL-1, and **(C)** IL-6 levels in the serum of the study mice. **(D)** Representative hematoxylin and eosin (H&E) staining of the colon (scale bar = 50 μm) and spleen (scale bar = 100 μm) sections from the mice. The colonic crypts are marked with black ellipses, and the inflammatory cell infiltration is indicated by arrows. *p < 0.05, **p < 0.01, and ****p < 0.0001 (vs. model).

To further investigate the protective effects of Clinodiside A, we performed ELISA and H&E staining to evaluate colonic tissue damage. As shown in Figures 2A–C, the serum levels of TNF-α, IL-1, and IL-6 were markedly elevated in the DSS-treated mice relative to the control group. H&E staining further revealed that Clinodiside A administration notably ameliorated DSS-induced crypt loss, inflammatory cell infiltration, and splenic injury, especially at the low treatment dose, demonstrating a particularly robust protective effect (Figure 2D); here, the colonic crypts are marked with black ellipses and inflammatory cell infiltration is indicated by arrows. The histological examination (Figure 2D) further confirmed that DSS induced obvious structural destruction of the colonic mucosa, as characterized by massive crypt loss and dense inflammatory cell infiltration in the mucosal layer. In contrast, Clinodiside A treatment, especially at the lower dose, alleviated these pathological injuries effectively by restoring the crypt structure and reducing inflammatory cell infiltration. These morphological changes are clearly distinguishable in the histological images and strongly support the protective effects of Clinodiside A on colonic tissue. In this work, we did not observe a dose-dependent effect of Clinodiside A, and the results showed that low-dose Clinodiside A was more effective than high-dose Clinodiside A.

### 3.2 Clinodiside A decreased iron levels and lipid peroxidation in DSS-induced mice

**FIGURE 3:**
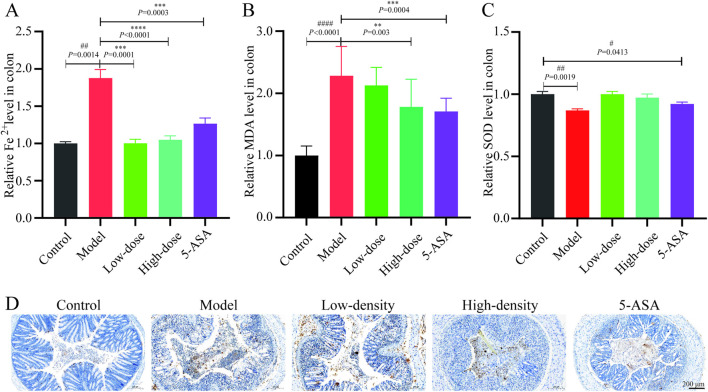
Clinodiside A reduces the elevated iron levels associated with DSS-induced colitis: relative **(A)** Fe^2+^, **(B)** malondialdehyde (MDA), and **(C)** superoxide dismutase (SOD) activity levels in the colon tissues of the study mice. **(D)** Representative pictures of the colon tissues of the study mice showing Prussian blue staining. Scale bar = 200 μm; ^#^ *p* < 0.05 (vs. control) and *p < 0.05 (vs. model).

To elucidate the potential mechanisms by which Clinodiside A ameliorates UC, colon tissue samples collected from all the mice were subjected to further analyses. Consistent with expectations, iron overload and lipid peroxidation manifested as elevated MDA level and diminished SOD activity (Figures 3A,B), respectively; these emerged as the key pathological features of intestinal epithelial injury in UC and are closely associated with ferroptosis-related cell death. Unexpectedly, low-dose Clinodiside A exhibited a greater capacity to reduce DSS-induced iron overload (approximately 46.62%, *p* = 0.0001) than high-dose Clinodiside A (approximately 44.15%, *p* < 0.0001, Figure 3A). Moreover, Clinodiside A treatment marginally reduced DSS-induced high MDA level (by approximately 6.75%, not significant, Figure 3B) and increased the SOD activity that was suppressed by DSS (Figure 3C). Consistently, Prussian blue staining demonstrated that DSS administration markedly increased iron accumulation in the colon, while Clinodiside A reduced such elevated iron levels in the colon (Figure 3D).

### 3.3 Clinodiside A alleviated DSS-induced damage in HCT116 cells

**FIGURE 4:**
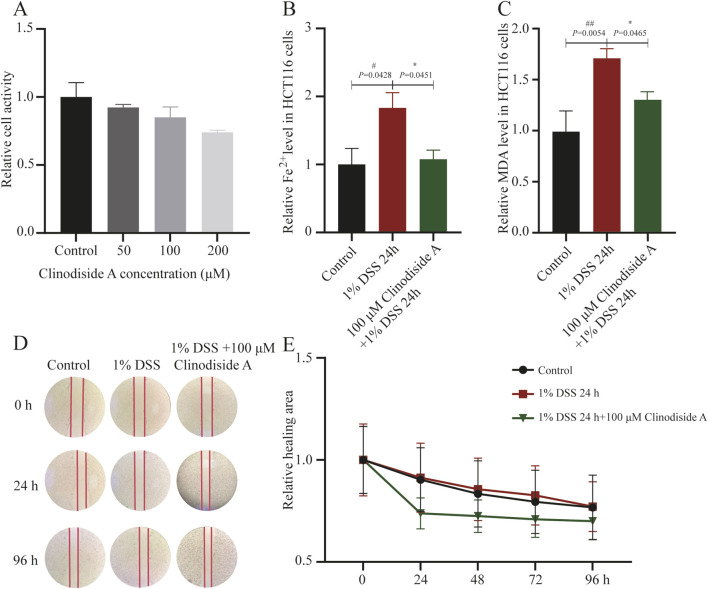
Clinodiside A alleviates DSS-induced damage in the HCT116 cells. **(A)** Relative activities of the HCT116 cells decrease as Clinodiside A concentrations (50–200 μM) increase. Relative **(B)** Fe^2+^ and **(C)** MDA levels in the HCT116 cells. **(D)** Representative images and **(E)** statistical results of the wound healing assay performed on the HCT116 cells. ^#^ *p* < 0.05 and ^##^ *p* < 0.01 (vs. control); *p < 0.05 (vs. model).

To further clarify the mechanisms underlying the protective effects of Clinodiside A against colitis-associated intestinal mucosal injury, we performed a cell migration assay of the impact of Clinodiside A on the migratory capacity of intestinal epithelial cells (IECs) (HCT116 cells) under colitic conditions. Our preliminary cytotoxicity assessment revealed that Clinodiside A had negligible toxicity on HCT116 cells at concentrations up to 200 μM (Figure 4A). An *in vitro* colitis model was subsequently established by exposing the HCT116 cells to 1% DSS for 24 h. Compared to the control cells, 1% DSS treatment for 24 h increased the iron (approximately 83.01%, *p* = 0.0428, Figure 4B) and MDA (approximately 71.9%, *p* = 0.0054, Figure 4C) levels in the HCT116 cells significantly. Consistent with the *in vivo* results, when the HCT116 cells were cotreated with 100 µM of Clinodiside A and 1% DSS for 24 h, the relative iron level reduced by approximately 41.18% from 1.8308 to 1.0769 (*p* = 0.0451, Figure 4B), and the MDA level reduced by approximately 23.62% from 1.719 to 1.3130 (*p* = 0.0465, Figure 4C). The wound healing assay on the HCT116 cells showed that although treatment with 100 µM of Clinodiside A could reduce the area of damage caused by 1% DSS, this change was not statistically significant (Figures 4D,E).

### 3.4 Clinodiside A treatment did not significantly damage the major organs in UC mice

**FIGURE 5:**
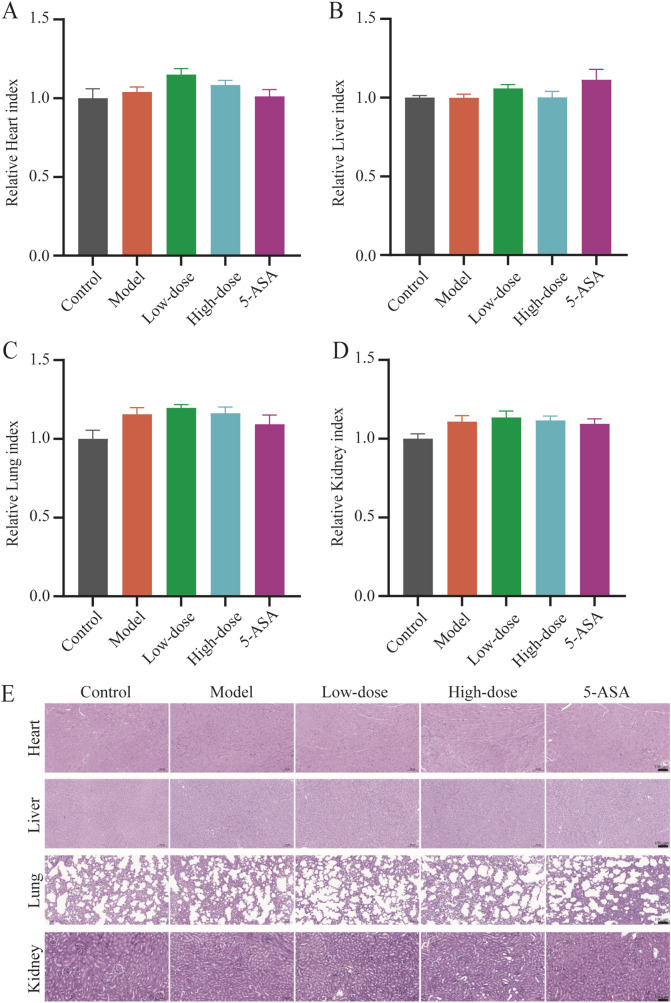
Clinodiside A treatment did not cause significant damage to the major organs in mice with UC. Clinodiside A has no effects on the relative **(A)** heart, **(B)** liver, **(C)** lung, and **(D)** kidney index scores of UC mice. **(E)** Representative pictures of H&E staining of tissues from the major organs of the study mice. Scale bar = 100 μm.

To evaluate the biosafety of Clinodiside A, we examined the major organs (heart, liver, lungs, and kidneys) of the mice in each group. We first weighed these organs for each group and calculated the corresponding relative organ index values; the results showed that Clinodiside A had no significant effects on the heart (Figure 5A), liver (Figure 5B), lungs (Figure 5C), and kidneys (Figure 5D). To further verify these results, we performed pathological examinations via H&E staining, which showed that Clinodiside A treatment did not cause significant damage to these major organs in UC mice (Figure 5E). Within the conditions of this study, we did not observe any obvious organ toxicity, which suggests the favorable short-term safety of Clinodiside A at the tested doses.

## 4 Discussion

The present study confirms that Clinodiside A effectively alleviates DSS-induced UC both *in vivo* and *in vitro*. Preliminary evidence from our study suggests that the protective effects of this active metabolite are associated with reduced iron overload, reduced lipid peroxidation, and enhanced intestinal epithelial repair rather than fully elucidating the ferroptosis mechanism. However, we only detected the indirect indicators (Fe^2+^, MDA, and SOD) related to ferroptosis, while the core ferroptosis markers (GPX4 and ACSL4) and signaling pathways (Nrf2/GPX4) were not validated. Therefore, the involvement of ferroptosis in the protective effects of Clinodiside A remains speculative and requires further molecular verification.

Clinodiside A (buddlejasaponin IVb) is a natural triterpenoid saponin that is ubiquitously distributed in diverse biological sources, including *Pleurotus ostreatus*, *C. chinense* (Benth.) O. Kuntze, and *Buddleja lindleyana* Fort., and serves as the active metabolite of the Chinese herba *C. chinense* known for its anti-inflammatory and hemostatic effects (Li et al., 2020). Notably, inflammation and bleeding are some of the hallmark clinical manifestations of UC, which is an incurable and widely prevalent chronic inflammatory disease. Wang et al. (2024) demonstrated that *C. chinense* Kuntze ameliorates DSS-induced UC in mice by reducing systemic inflammation and modulating the metabolism. However, the specific component responsible for this protective effect remains to be elucidated as Clinopodii herba contains a wide array of bioactive compounds, such as luteolin-7-O-glucoside, hesperidin, quercetin, buddlejasaponin IVb, rosmarinic acid, and buddleoside (Montgomery et al., 2025). Hence, we studied Clinodiside A as the active metabolite of Clinopodii herba that exhibits anti-UC effects by effectively reducing the inflammatory responses as well as elevating the iron and MDA levels in the UC model; these findings are consistent with the results reported in literature (Wang et al., 2023). Thereafter, Li et al. (2024) also found that Clinodiside A (buddlejasaponin IVb) improved motor dysfunction in Parkinson’s disease by suppressing iron-overload-mediated dopaminergic neuron ferroptosis, which further confirmed our results. Thus, we aimed to explore the mechanisms by which Clinodiside A modulates iron overload and lipid peroxidation in UC, which may ultimately contribute to its anti-inflammatory and mucosal repair effects. Wu et al. (2024) were the first to report in 2024 that buddlejasaponin IVb alleviates DSS-induced UC by inhibiting ferroptosis mediated by the Nrf2/GPX4 pathway; these findings also confirm our current results.

To this end, we also performed *in vitro* validation on a DSS-treated HCT116 cell model of UC (Liang et al., 2025) to ensure consistency with the *in vivo* experiments. First, the results showed that Clinodiside A reduced the DSS-induced elevation of iron and MDA levels in the HCT116 cells, which were consistent with the *in vivo* results. Second, given that continuous and diffuse colonic mucosal ulceration is a defining feature of UC, we examined the effects of Clinodiside A on wound healing using a cell scratch assay. Clinodiside A promoted wound closure in the HCT116 cells, thereby facilitating intestinal barrier repair. Crucially, this pro-healing effect has not been reported in previous studies (Wang et al., 2023; Wu et al., 2024). Therefore, our findings uncover an additional possible mechanism by which Clinodiside A exerts its anti-UC effects.

The concentrations of Clinodiside A used in this study were 10 mg/kg and 40 mg/kg, which were different from those used by Wu et al. (2024); in addition, the administration method and duration were different. Wu et al. (2024) administered Clinodiside A for a total of 14 d along with a 7-d pretreatment period before DSS induction; however, our treatment duration was 21 d with a 14-d pretreatment period. Wu et al. (2024) also showed that treatment with increasing concentrations (5, 10, and 20 mg/kg) of buddlejasaponin IVb improved the anti-UC effects; however, no dose-dependent effects were observed in our study, while the low-dose (10 mg/kg) Clinodiside A treatment showed better efficacy. This suggests a non-linear dose–response relationship that may be caused by target saturation, altered pharmacokinetics, or mild adverse effects at excessively high concentrations. The underlying reason for this variation needs further investigation. Additionally, as safety evaluations, we performed pathological examinations on the major organs of the mice, which showed that 10 mg/kg/d and 40 mg/kg/d of Clinodiside A for 21 d did not cause damage to the body; this indicates that Clinodiside A has good biosafety.

Notwithstanding the encouraging findings, this study also has some limitations. HCT116 is a cancer cell line with altered migration and metabolism, which may not fully replicate the physiological state of normal IECs. Although the HCT116 cell line has been widely used in UC-related *in vitro* studies, further verifications using non-tumorigenic IECs, such as NCM460 or Caco-2, or intestinal organoids are needed to confirm our *in vitro* findings (Li et al., 2023; Kawaguchi et al., 2023; Luthra et al., 2022).

## 5 Conclusion

Conclusively, the present study reveals the protective mechanisms of Clinodiside A in DSS-induced UC models, such as anti-inflammatory effects, iron metabolism regulation, and lipid peroxidation reduction. Moreover, the *in vitro* wound healing assay provides preliminary evidence for the potential role of Clinodiside A in intestinal epithelial repair. These findings provide a theoretical basis for the development of Clinopodii herba as a therapeutic agent against UC, in addition to suggesting a plausible approach for the treatment of UC.

## Data Availability

The original contributions presented in the study are included in the article/supplementary material; further inquiries may be directed to the corresponding author.
